# Ultrafast Photoinduced Dynamics in 1,3-Cyclohexadiene: A Comparison of Trajectory Surface Hopping Schemes†

**DOI:** 10.1021/acs.jctc.4c00012

**Published:** 2024-07-01

**Authors:** Edison X. Salazar, Maximilian F. S. J. Menger, Shirin Faraji

**Affiliations:** † Instituut-Lorentz, Universiteit Leiden, 2300 RA Leiden, The Netherlands; ‡ Theoretical Chemistry, Zernike Institute for Advanced Materials, University of Groningen, Nijenborgh 4, 9747 AG Groningen, The Netherlands; § Theoretische Chemie, Physikalisch-Chemisches Institut, Universität Heidelberg, 69120 Heidelberg, Germany; ⊥ Institute of Theoretical and Computational Chemistry, Faculty of Mathematics and Natural Sciences, Heinrich Heine University Düsseldorf, 40225 Düsseldorf, Germany

## Abstract

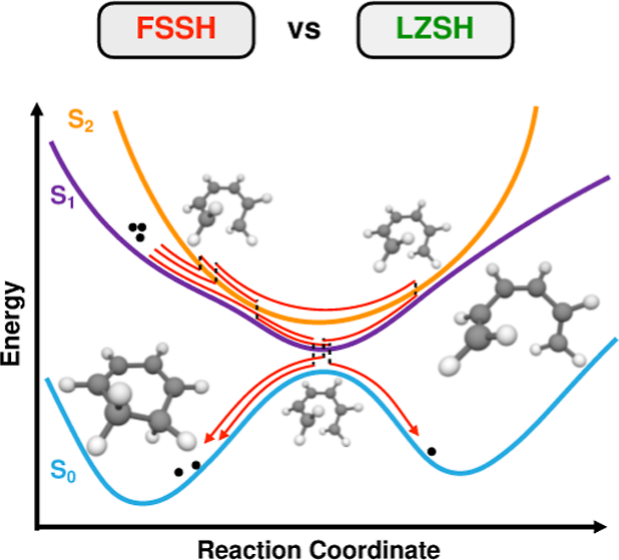

Photoinduced nonadiabatic processes play a crucial role in a wide range of disciplines, from fundamental steps in biology to modern applications in advanced materials science. A theoretical understanding of these processes is highly desirable, and trajectory surface hopping (TSH) has proven to be a well-suited framework for a wide range of systems. In this work, we present a comprehensive comparison between two TSH algorithms, the conventional Tully’s fewest switches surface hopping (FSSH) scheme and the Landau–Zener surface hopping (LZSH), to study the photoinduced ring-opening of 1,3-cyclohexadiene (CHD) to 1,3,5-hexatriene at the spin-flip time-dependent density functional theory (SF-TDDFT) level of theory. Additionally, we compare our results with a literature study at the extended multistate complete active space second-order perturbation theory method (XMS-CASPT2) level of theory. Our results show that the average population and lifetimes estimated with LZSH using SF-TDDFT are closer to the literature (using multireference methods) than those estimated with FSSH using SF-TDDFT. The latter speaks in favor of applying LZSH in combination with the SF-TDDFT method to study larger and more complex systems such as molecular photoswitches where the CHD molecule acts as a backbone. In addition, we present an implementation of Tully’s FSSH algorithm as an extension to the PySurf software package.

## 1 Introduction

Photoinduced processes play an important role in sustaining the everyday life of human beings on earth. For example, they are present in the fundamental steps of photobiological processes such as vision or the synthesis of vitamin D in the skin.^1,2^ They are also critical to the development of new technologies, such as molecular electronic devices that can be controlled by light.^3,4^ The photoinduced processes are governed mainly by nonadiabatic transitions,^5−7^ also called non-Born–Oppenheimer (BO) transitions,^8^ which are radiationless electronic transitions between different BO (or adiabatic) electronic states along the dynamic of a chemical reaction. These transitions are characterized by comparable time scales for electronic and nuclear motions and a small energy gap between different electronic potential energy surfaces (PESs).^9−11^ When the energy gap is (approximately) zero, the system is prone to breakdown in the BO approximation, leading to crossings or conical intersections (CIs) between the PESs.^11,12^

In the last few years, trajectory surface hopping (TSH) simulations have proven to be a powerful tool for the understanding and exploration of photoinduced processes.^11,13,14^ In the TSH methods, the nuclear motion of the system is approximated by a swarm of independent classical trajectories. Each trajectory is propagated on an active (adiabatic) electronic surface. Unlike in BO dynamics, the active state can change to another electronic state, thereby including nonadiabatic transitions.^14−16^ The most widely used algorithm is fewest switches surface hopping (FSSH) developed by Tully.^17,18^ In this approach, the active state of each trajectory is determined according to the computed state probabilities using a minimum number of hops between electronic states.^11,17^ Despite its straightforward implementation, the low computational cost compared to that of nonadiabatic quantum molecular dynamics methods,^13,14,19^ and its success in obtaining qualitative and quantitative information on numerous photochemical mechanisms, FSSH lacks internal consistency,^20,21^ i.e., the average of the electronic population and the fraction of trajectories in each state are not the same. Ad hoc decoherence corrections can eliminate this problem.^22−24^ Within the framework of TSH methods, the Landau–Zener surface hopping (LZSH) approach^25−27^ is a simpler and computationally cheaper method to perform nonadiabatic dynamics.^28−31^ Unlike the FSSH method, which requires solving the time-dependent electronic Schrödinger equation and computing nonadiabatic couplings (NACs) or wave function overlaps at each time step, the LZSH method requires only energy differences between two electronic states. Therefore, LZSH does not account for decoherence corrections and is computationally more efficient.^28,29^ These features make LZSH attractive for electronic structure methods where NACs are computationally expensive or not implemented.^30^ Recently, Xie et al.^28,29^ have shown that nonadiabatic dynamics simulations with FSSH and LZSH give similar results on the photodissociation of phenol and pyrazine molecules. Additionally, Yue et al.,^32^ using a variant of LZSH called Zhu–Nakamura surface hopping,^33^ have shown that both methods deliver similar qualitative results on the photoisomerization process of the cis/trans-azobenzene. These findings suggest the potential utility of LZSH in exploring more complex photochemical processes. On the other hand, Polyak et al.,^34^ in a comprehensive theoretical study on the 1,3-cyclohexadiene (CHD) photodissociation using the extended multistate complete active space second-order perturbation theory (XMS-CASPT2^35^) method, pointed out that a complex nonadiabatic topography like in this reaction requires a more general algorithm, such as FSSH with decoherence correction. However, recently, in a similar study on CHD, Zhang et al.^36^ obtained closer results to the experimental ones^37^ than Polyak et al. using XMS-CASPT2 in combination with the curvature-driven coherent switching with decay of mixing (κCSDM) dynamical method.^38^ The latter method requires only energies and gradients. In this context, there is a lack of studies on an extensive comparison between LZSH and FSSH on an equal footing for large molecules.^39^

**Figure 1:**
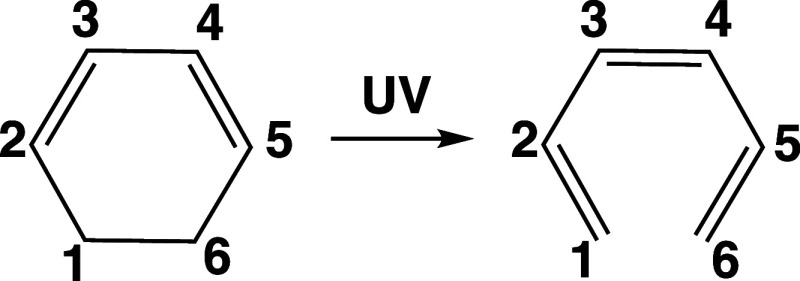
Chemical structures of CHD (left side) and HT (right side).

The photochemical interconversion between CHD and 1,3,5-hexatriene (HT), Figure 1, is one of the most widely studied nonadiabatic processes, both theoretically^34,36,40−52^ and experimentally.^37,43,44,49,53−55^ This (4n + 2) photoelectrocyclic reaction serves as a simplified model for studying the photophysical and photochemical properties of important macromolecules based on the CHD chromophore.^43,44^ The interconversion of CHD (isolated) occurs through at least two CIs, and a third CI has been recently proposed as an alternative deactivation path.^36,47^ This reaction mechanism offers an attractive and complex scenario to compare FSSH and LZSH using a similar level of theory. Our recent theoretical study^47^ on this photochemical process using spin-flip time-dependent density functional theory (SF-TDDFT^56^) has successfully described and characterized the most important geometries on the PESs along the ring-opening/closure reaction coordinate, in agreement with those obtained by multireference wave function methods. Our benchmark calculations on the first two excited states of the CHD reveal that SF-TDDFT, in particular, in combination with the BHHLYP functional, shows reasonable performance compared to wave function-based methods and experimental results, suggesting that SF-TDDFT could be a good low-cost method to study complex molecules that contain the CHD chromophore^57^ as their photochemically active backbone. Thus, the present work contributes to this ongoing discussion by comparing the accuracy and capability of the FSSH and LZSH methods at the SF-TDDFT level to capture the main features of the nonadiabatic dynamics associated with the photoisomerization process of the CHD molecule. In addition, we present an implementation of Tully’s FSSH algorithm as an extension to the PySurf software package.^30^ PySurf is a platform based on Python that simplifies the process of method development by providing solid and extensible core functionalities. The Plugin engine guarantees its modularity and allows for new modules, such as the FSSH implementation presented here, to be easily added. This means that both users and developers can effortlessly test new ideas and implementations in PySurf with a low entry barrier. In addition, PySurf provides a database nature that allows the usage and implementation of several interpolation schemes as well as machine learning-based approaches. Here, we introduce the FSSH plugin implementation, its comparison, and its performance with LZSH benchmarked with a well-studied system, i.e., CHD.

This work is organized as follows: Section 2 briefly describes the underlying theoretical methods used in this work. Sections 3 and 4 describe the implementation of the FSSH method and the computational details, respectively. Our results and discussion are presented in Section 5, and finally, our concluding remarks are given in Section 6.

## 2 Theory

### 2.1 Trajectory Surface Hopping

In recent years, TSH has emerged as an effective scheme to study the nonadiabatic dynamics of small to intermediate molecular systems. Due to its simple approach and the use of independent semiclassical trajectories, TSH has become a popular nonadiabatic molecular dynamics method.^13,14,19^ Several variants of TSH have been developed over the years.^14^ Here, we briefly review Tully’s fewest switching and the LZSH approaches.

In all TSH approaches, the nuclei are propagated on a single (adiabatic) electronic PES using classical equations of motion given by Newton’s second law: *m*_α_**a**_α_ = **f**_α_, with *m*_α_ being the mass of atom α, **a**_α_ the acceleration, and **f**_α_ the force. A suitable algorithm to integrate this equation of motion is the velocity Verlet algorithm,^58^ where the positions and the velocities are computed at the same time step. The way surface hopping incorporates nonadiabatic effects is by allowing changes in the active electronic PES, so-called hops. Thus, the dynamics of the nuclei is influenced by multiple electronic states. When a hop occurs, the potential energy at that position changes instantaneously. To ensure the total conservation of energy, the velocities must be rescaled. This involves conversion between potential and kinetic energy. Typically, velocities are rescaled according to one of the following schemes
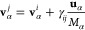
1
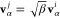
2where **u**_α_ is a vector adjusted by a scalar factor γ_*ij*_ and β is a positive factor determined by the total energy conservation, *T* + *H*_*ii*_ = β^2^*T* + *H*_*jj*_, with *T* and *H* being the kinetic and potential energy, respectively. The velocities are rescaled along only the adjustment vector. The selection of the **u**_α_ vector defines another parameter for TSH, where a common choice is the NAC vector. If the latter is not implemented or too expensive to compute, the **u**_α_ vector can be defined by the momentum.^59^

### 2.2 FSSH Probability

In the FSSH, in addition to nuclear propagation, the electronic degrees of freedom are also propagated. The latter follows the time-dependent Schrödinger equation
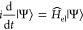
3where  is the electronic Hamiltonian. All equations are in atomic units, i.e., *ℏ* = 1. To solve eq 3, the electronic wave function is typically expanded in terms of the adiabatic basis using ∑_*i*_|ψ_*i*_⟩⟨ψ_*i*_| = *I*
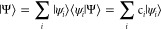
4

Substituting eq 4 into eq 3 and projecting on ⟨ψ_*j*_|, one obtains the following equation of motion for the expansion coefficients *c*_*i*_
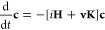
5where the coefficient vector **c** contains the elements ⟨ψ_*i*_|Ψ⟩, **H** is the Hamiltonian matrix, and **K** is the NAC matrix. Notice that the electronic Hamiltonian matrix **H** in eq 5 is diagonal on the adiabatic basis. However, in the case of spin–orbit coupling (relevant for intersystem crossing), the Hamiltonian matrix can have nonzero off-diagonal elements given by the spin–orbit coupling of the interaction between singlet and triplet electronic states. For such a scenario, González et al.^16,60,61^ have proposed a general method that diagonalizes the total Hamiltonian matrix (electronic Hamiltonian plus an additional term responsible for the spin–orbit coupling) by choosing a different basis that is obtained by the unitary transformation from the electronic Hamiltonian basis.

With the coefficients and their time propagation given, the hopping probability can be computed in Tully’s FSSH algorithm as
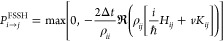
6where the maximum value is taken to avoid negative probability values. To determine whether the trajectory hops from the current state to another state, the computed probability is compared with a random number between 0 and 1 using the following criteria
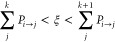
7

A drawback associated with FSSH is overcoherence.^14,21,24,62^ Over the years, different decoherence correction schemes have been developed to address this problem.^14,63^ One simple and practical correction to the decoherence problem was proposed by Granucci and Persico.^24^ In this approach, a nonlinear decay of mixing model (a simplified version of the Truhlar et al. models^22,23^) is applied at each time step, transforming the coefficients as follows
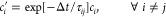
8
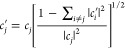
9
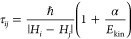
10where the empirical parameter α = 0.1 hartree and *E*_kin_ is the nuclear kinetic energy.

### 2.3 Landau–Zener Surface Hopping

In LZSH, in contrast to FSSH, the hopping probability is a function of the energy gap between two adiabatic PESs (Δ*H*_*ij*_ = |*H*_*i*_ – *H*_*j*_|). In the adiabatic form, the hopping probability^64^ is written as follows
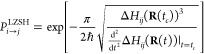
11where **R**(*t*) is the nuclear position evaluated at the time *t*_*c*_ when the energy gap achieves its minimum, i.e., if in a sequence of three timesteps, the following is satisfied: Δ*H*_*ij*_(**R**(*t* – Δ*t*)) > Δ*H*_*ij*_(**R**(*t*)) and Δ*H*_*ij*_(**R**(*t*)) < Δ*H*_*ij*_(**R**(*t* + Δ*t*)). Notice that the electron propagation is not needed to compute the hopping probability; thus, LZSH is free from decoherence issues. Therefore, the hopping probability for LZSH is computed only when the adiabatic energy gap attains its minimum, while the hopping probability for FSSH is computed at each time step along a trajectory. Similar to FSSH, the computed hopping probability is compared with a random number generated from 0 to 1 to determine whether the trajectory hops from state *i* to state *j* or not.

## 3 Implementation

**Figure 2:**
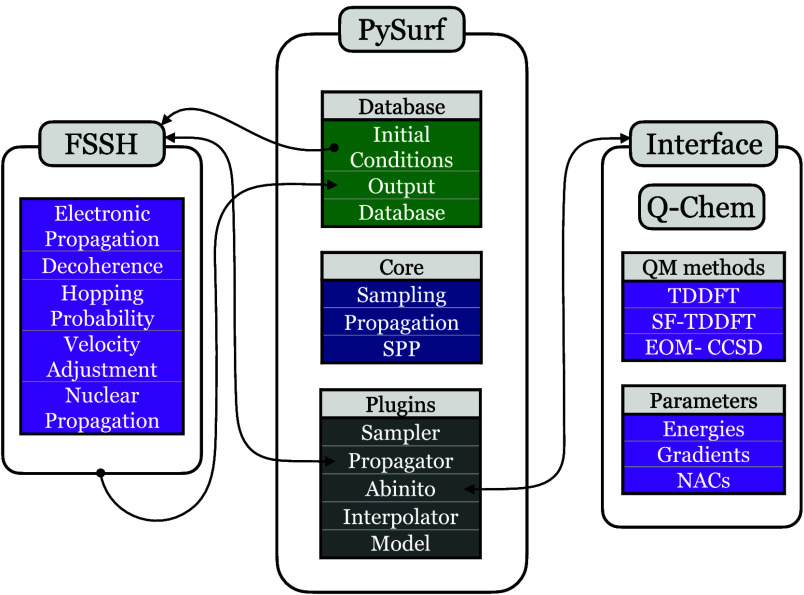
Schematic description of the code structure of the implemented FSSH algorithm, PySurf, and the interface to Q-Chem. SPP stands for surface point provider and is responsible for providing the properties (energies, gradients, and NACs) in a standardized data format at a given geometry.^30^

The FSSH algorithm is implemented as a propagator in PySurf, a new framework for computational chemistry calculations.^30^ PySurf is written in the Python programming language (Python3.6+) and originally comes with support for LZSH. The package has a powerful Plugin engine that allows the extension of the core package. Figure 2 shows a schematic description of the basic steps of the FSSH method implemented and the interface communication with PySurf and Q-Chem.^65^ The FSSH Plugin is based on the standard FSSH algorithm proposed by Tully^17^ plus a decoherence correction to avoid overcoherence^15^ and a velocity adjustment to conserve the total energy. The FSSH Plugin workflow is arranged in the following steps: **(1)** the initial conditions [**R**(*t*_0_), **V**(*t*_0_), and **C**^adi^(*t*_0_)] for the trajectories are generated using a sampler (Wigner or normal mode), **(2)** the parameters of the dynamics and the electronic structure calculations for the input files for each trajectory are set up, **(3)** the dynamics simulation is launched, and **(4)** the analysis of results is saved in the database. The FSSH Plugin is publicly available.^66^

## 4 Computational Details

Nonadiabatic dynamics simulations were performed using our newly implemented FSSH algorithm^66^ and the LZSH algorithm.^30^ Initial geometries and velocities were sampled by using a Wigner distribution at the ωB97X-D/cc-pVDZ level of theory. A set of 300 trajectories were launched and propagated from the first excited state (no energy window was imposed) for 400 fs using a time step of 0.5 fs for FSSH and LZSH. Notice that the initial condition sampling settings do not specify any particular energy window. The electronic propagation is computed using the exponential operator method^61,67^ by taking half of the time step. The FSSH calculations were performed with the decoherence correction, as explained in detail in Section 2. In both methods, the nuclear velocity vector is rescaled to ensure that the total energy is conserved after a transition. In the FSSH method, the velocity is changed in the direction of the NAC vector, as described in eq 1. If the velocity adjustment does not compensate for the change in potential energy induced by the hopping event, the hop is forbidden, and the system remains in its initial electronic state with the nuclear velocity reversed. Unlike FSSH, the total energy in LZSH is conserved by adjusting the velocity in the direction of the momentum, as shown in eq 2. Also, in LZSH, we do not consider any forbidden hopping events. Each simulation is performed using the SF-TDDFT(BHHLYP)/cc-pVDZ level of theory via an interface in PySurf^30^ with the electronic structure package Q-Chem.^65^ We chose the BHHLYP functional based on previous benchmark calculations^47,57^ on the photochemistry of CHD, which reveal that SF-TDDFT, in particular, in combination with the BHHLYP functional, shows good performance compared to wave function-based method^34^ and experimental results.^54^ One of the challenges of using SF-TDDFT in nonadiabatic dynamics is dealing with spin contamination, especially when the system moves further away from the Franck–Condon region.^68,69^ Thus, it is difficult to distinguish between single and triplet states without using a procedure to track the states. The criterion used by default to classify the states is to compute the  value. When , the states are assigned as singlet states. Over the past few years, there have been noticeable advancements in the development of SF-TDDFT versions that are free of spin contamination. These include the fully spin-adapted SF-TDDFT method^68,70,71^ and the mixed-reference SF-TDDFT method.^72,73^ However, despite their potential, these methods have not yet been widely adopted.

In this work, we used the  value to track the first three electronic singlet states (the reaction involved the first three electronic states) of a set of four states at each time step. If a trajectory shows more than two electronic states with high spin contamination , then the trajectory stops. Therefore, to estimate the average population of each electronic state, the surviving trajectories are averaged at each time step.

## 5 Results and Discussion

### 5.1 Spectrum

**Figure 3:**
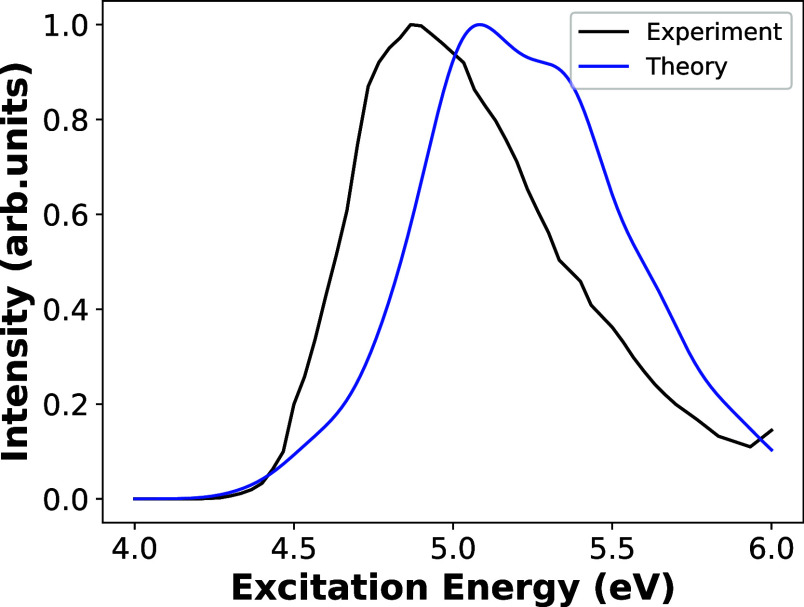
Normalized absorption spectrum of CHD (blue) computed at the SF-BHHLYP/cc-pVDZ level of theory using 300 geometries sampled from a Wigner distribution around the Franck–Condon point. Experimental data (black) are taken from ref (53). The absorption spectrum was obtained as a normalized superposition of Gaussians localized at computed excitation energies with a spectral broadening of 0.1 eV.

The absorption spectrum of CHD is depicted in Figure 3, where a computed spectrum and the experimental UV spectrum from the literature^53^ are compared. The absorption spectrum is computed for 300 geometries, taken from a Wigner distribution, using SF-BHHLYP/cc-pVDZ. The two lowest singlet electronic transitions contribute to the calculated absorption spectrum, i.e., from S_0_ → S_1_ and S_0_ → S_2_, where the brightest transition is from S_0_ → S_1_. The comparison between theory and experiment shows a very satisfactory qualitative and partly quantitative agreement. Notice that in the computed spectrum, the peak is blue-shifted by about 0.25 eV compared to the experimental peak. This difference in energy from the experimental value lies within the error range (0.1–0.5 eV) of TDDFT.^74^

### 5.2 Photoisomerization of CHD

Nonadiabatic dynamics simulations were performed using the SF-TDDFT(BHHLYP)/cc-pVDZ level of theory for 400 fs with a time step of 0.5 fs for two different TSH algorithms: FSSH and LZSH. We considered the simulation time and the time step based on previous theoretical works.^34,46^ By the end of the simulation time, the quantum yield (QY) for HT formation is calculated considering all the trajectories whose geometries show a bond distance between the reactive carbons C_1_–C_6_ (see Figure 1) larger than or equal to 3 Å. These results are summarized in Table 1. Figure 5a,b shows the population dynamics of the three lowest adiabatic electronic states: S_0_ (blue line), S_1_ (orange line), and S_2_ (green line), obtained with the FSSH and LZSH algorithms, respectively.

**Table 1:** Parameters of the Monoexponential Fit of S_1_ Population and QY Formation of HT^a^

methods	τ_d_ (fs)	τ_e_ (fs)	lifetime	QY(HT)
FSSH-SF-BHHLYP/cc-pVDZ	17 ± 2	116 ± 1	133 ± 2	35 ± 5
LZSH-SF-BHHLYP/cc-pVDZ	12 ± 2	84 ± 1	96 ± 2	42 ± 6
FSSH-XMS-CASPT2/cc-pVDZ^34^	12 ± 2	72 ± 9	84 ± 9	47 ± 8
κCSDM-XMS-CASPT2/def2-TZVP^36^		96		40
experiment^37^		230 ± 30		30

a τ_d_ represents the latency time and τ_e_ is the time constant for the population decay.

**Figure 4:**
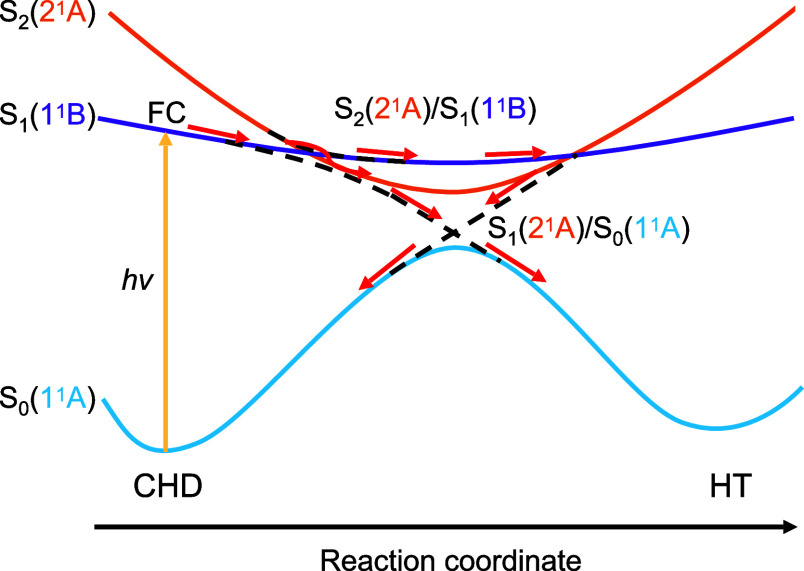
Schematic representation of the photochemical interconversion from CHD to HT. FC stands for the Franck–Condon region. Dashed lines indicate the crossing between excited states S _2_(2^1^ A) and S _1_(1^1^ B) and S _1_(2^1^ A) and the ground state S _0_(1^1^ A).

**Figure 5:**
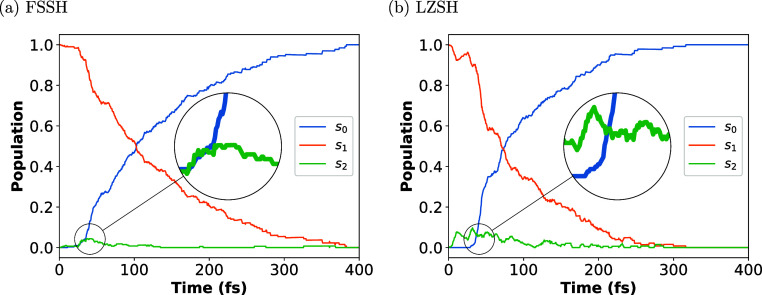
Population dynamics of the three lowest adiabatic electronic states (S_0_ in blue, S_1_ in orange, and S_2_ in green) involved in the photoisomerization process of the CHD molecule. Two different TSH algorithms were used: (a) the conventional Tully’s FSSH, and (b) the LZSH. Magnified circles show the peak of the maximum population transfer from S_1_ → S_2_.

Comparing the FSSH and LZSH results, we observe that in both cases, a fast population transfer takes place from the S_1_ to the S_2_ adiabatic states, where the maximum population transfer is achieved at 43 fs for FSSH and at 30 fs for LZSH. Additionally, it is observed that the S_0_ population begins to grow at 11 fs for FSSH and 30 fs for LZSH. Although the growth in FSSH begins earlier, it is a bit slower than its counterpart in LZSH. Similar maximum and time scales on S_2_ and S_0_ for LZSH were reported by Polyak et al.^34^ using FSSH with the XMS-CASPT2 method. They mention that this maximum (or peak) corresponds to the proportion of the wavepacket that gradually changes the state character of the adiabatic state S_1_ from 1^1^B to 2^1^ through the CI seam between S _1_(1^1^ B) and S _2_(2^1^ A)^b^ (see Figure 4). Additionally, they report two more peaks around 95 and 138 fs before the S_2_ population decays to zero by 200 fs. In our results, we observe a slower decay of the S_2_ state after the maximum population transfer is achieved in both algorithms, being fully zero at 369 and 316 fs for FSSH and LZSH, respectively. It is clear that LZSH shows more population transfer from S_1_ to S_2_ than does FSSH along the simulation time. The low population transfer from S_1_ to S_2_ in the first 60 fs for FSSH is observed even when the time step is reduced to 0.1 fs (see Figure S1 in Supporting Information and Figure 5a). The reason for the difference in population transfer is that FSSH incorporates the condition to avoid hops (also called frustrated hops) where the velocity in the direction of the NAC vector is not enough to conserve the total energy.^62^ Thus, the molecule remained in its current state. If this condition is not satisfied, we observe a substantial increase of hops between S_1_ and S_2_ in FSSH as well as a reduction in the QY formation of HT (see Figure S2a,b and Table S1 in Supporting Information). Interestingly, if we consider in LZSH the scenario where after a hop the NAC vector is computed and the velocity is rescaled in the direction of that vector as FSSH, i.e., considering frustrated hop constraints, we observe that LZSH under these conditions shows less population transfer from S_1_ to S_2_ than normal LZSH. This behavior is similar to FSSH (see Figure S4 in Supporting Information). Comparing the evolution of the population of the S_0_ and S_1_ states for both algorithms, we observe clearly that after a time scale of about 30 fs, the S_1_ population decays faster in the case of LZSH (being zero at 317 fs) than FSSH (being zero at 383 fs). Likewise, the S_0_ population grows faster in the case of LZSH (being 100% at 317 fs) than in the case of FSSH (being 100% at 383 fs). We estimated the lifetime of the S_1_ population by fitting the following monoexponential function
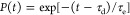
12where τ_d_ and τ_e_ correspond to the latency time and the time constant for the population decay, respectively. These parameters are listed in Table 1. In general, FSSH predicts a longer lifetime than LZSH. Notice that the τ_d_ and τ_e_ predicted with LZSH are in agreement with those obtained by Polyak et al.^34^ Additionally, the τ_e_ is in agreement with the 96 fs predicted by Zhang et al.^36^ In the case of FSSH, particularly for the time constant τ_e_, it shows a close value to the experimental result.^37^ Furthermore, by comparing the QY formation of HT, we see that FSSH computes the closest value to the experiment. Although LZSH delivers a QY far from the experimental one, this QY agrees with those computed with the XMS-CASPT2 method. We found this fact particularly interesting since our main goal is to compare LSZH and FSSH at the same level of electronic structure theory.

**Figure 6:**
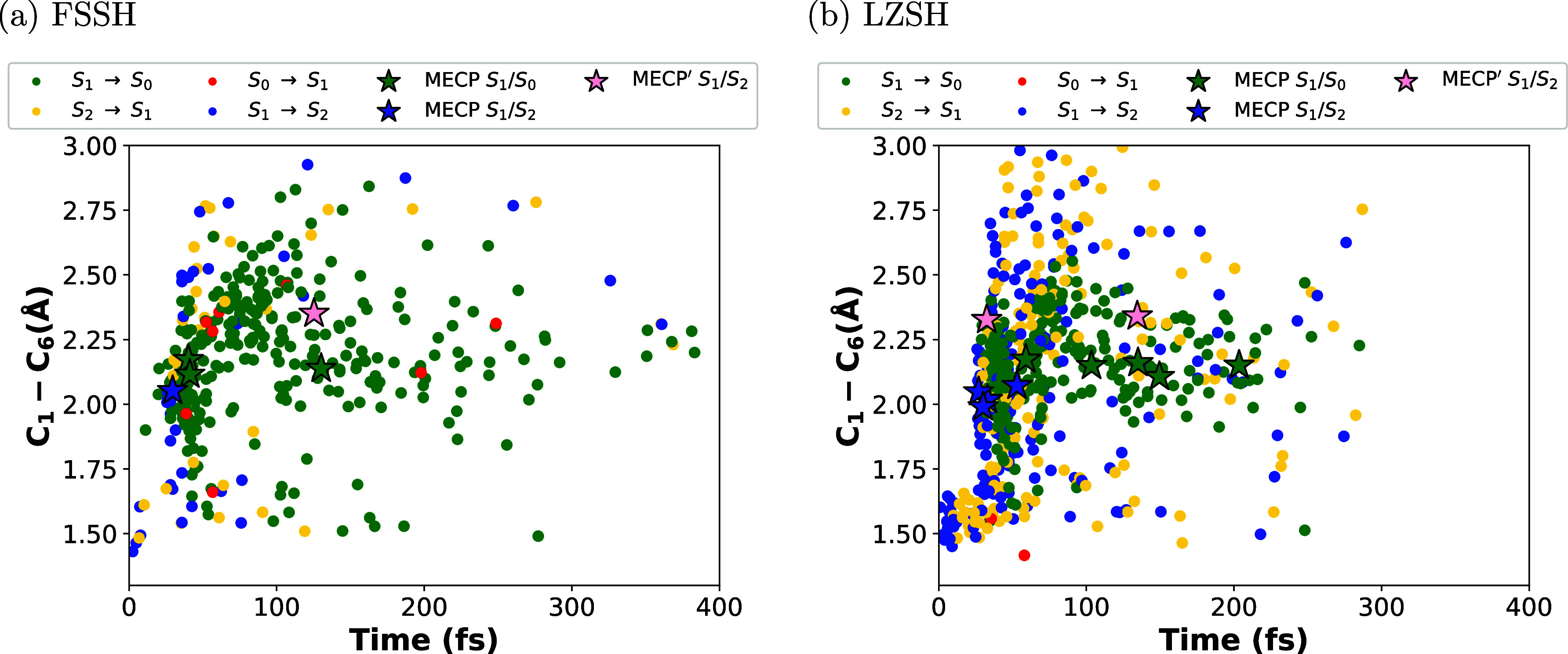
Distribution of molecular geometries along the simulation time (400 fs) for the C_1_–C_6_ distance for 300 trajectories. Transitions are described for the full-colored circles and stars labeling the MECPs for S_1_ → S_2_ and S_1_ → S_0_. Panels (a,b) stand for the conventional Tully’s FSSH and the LZSH, respectively.

**Figure 7:**
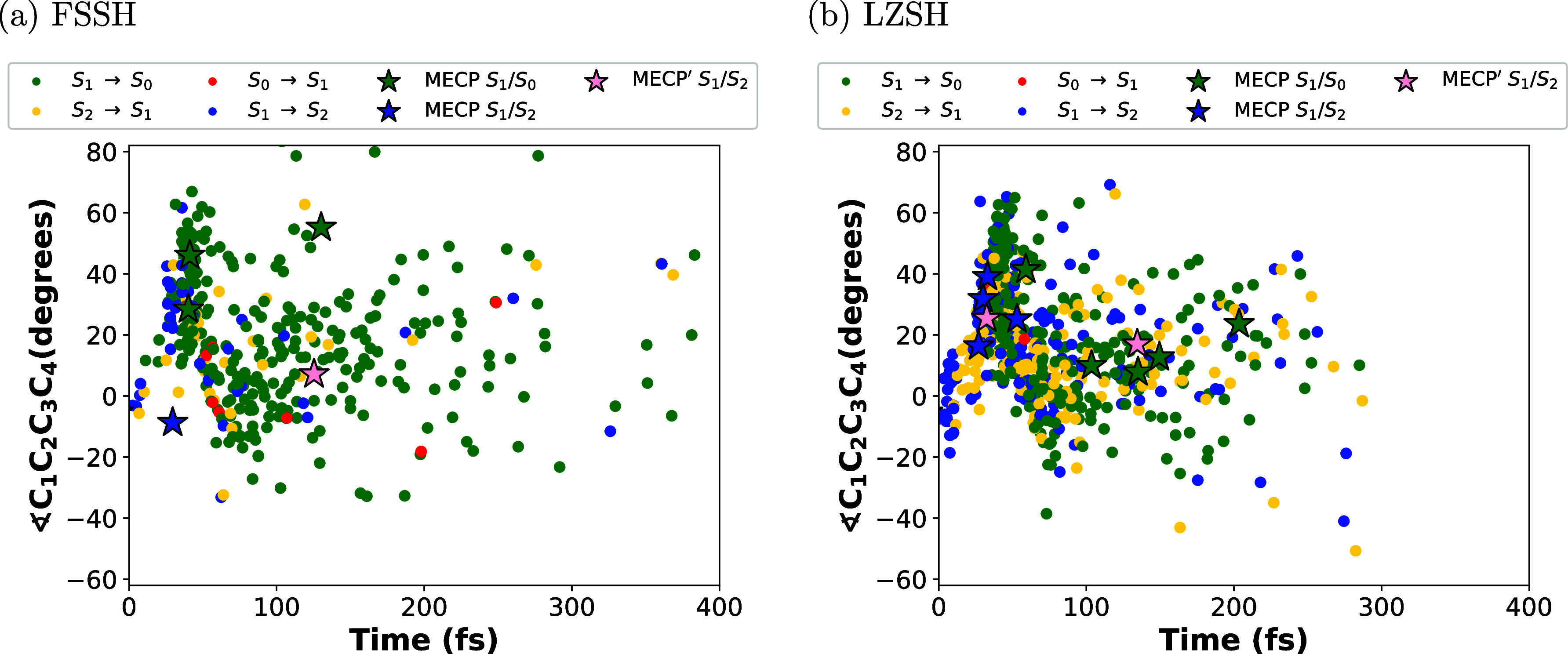
Distribution of molecular geometries along a simulation time (400 fs) for the torsion angle ∠C_1_C_2_C_3_C_4_ for 300 trajectories. Transitions are described for the full-colored circles and stars labeling the MECPs for S_1_ → S_2_ and S_1_ → S_0_. Panels (a,b) stand for the conventional Tully’s FSSH and the LZSH, respectively.

**Figure 8:**
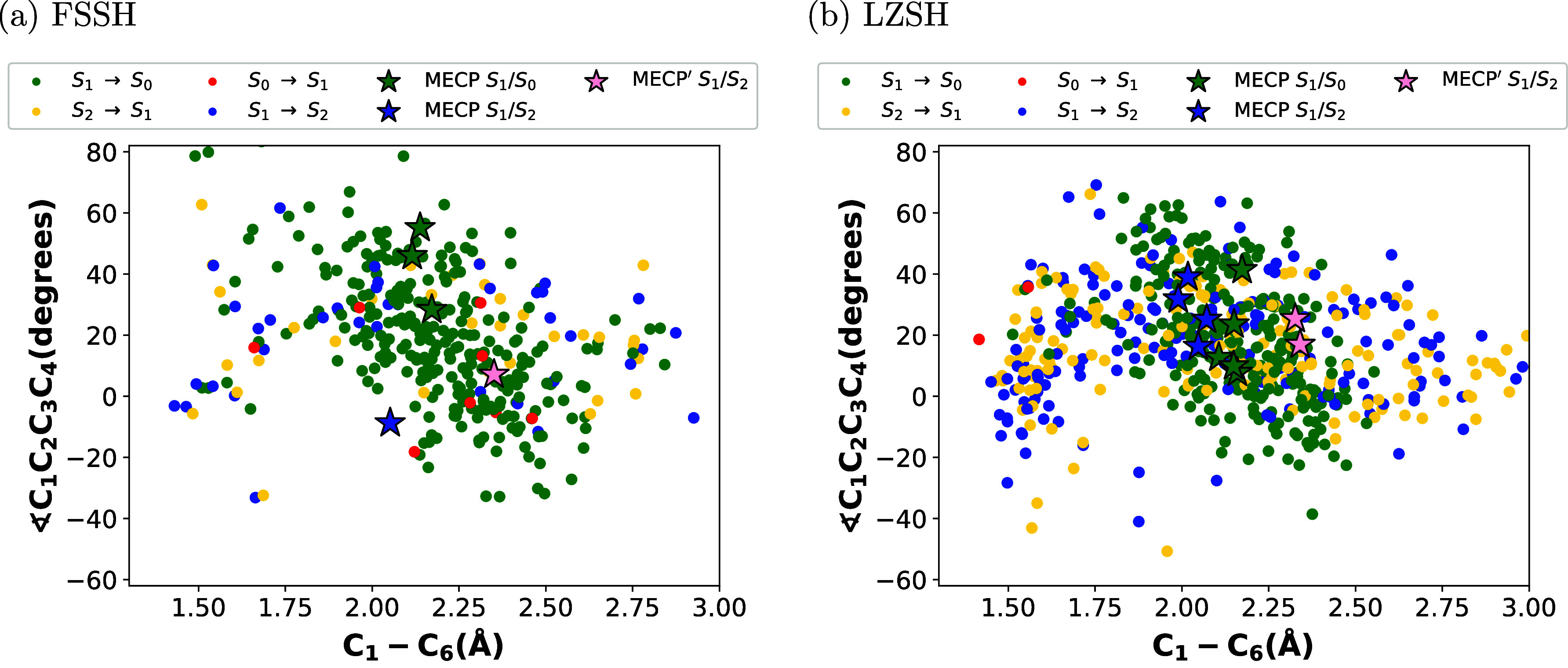
Distribution of molecular geometries along the bond-breaking coordinate and the torsion angle ∠C_1_C_2_C_3_C_4_ for 300 trajectories. Transitions are described for the full-colored circles and stars labeling the MECPs for S_1_ → S_2_ and S_1_ → S_0_. Panels (a,b) stand for the conventional Tully’s FSSH and the LZSH, respectively.

Similar to the plots made by Polyak et al.^34^ for the distribution of geometries corresponding to transitions between the three electronic states involved in the reaction and the trajectory density heat maps, we shall illustrate our comparison between FSSH and LZSH for the photoisomerization of CHD. Figures 6–8 depict the distribution of the transitions between pairs of electronic states from those involved in the reaction. These transitions are described by full-colored circles. Therefore, the full-blue circle is S_1_ → S_2_, the full-yellow circle is S_2_ → S_1_, the full-green circle is S_1_ → S_0_, and the full-red circle is S_0_ → S_1_. Each figure is the plot of the C_1_–C_6_ distance vs time, the torsion angle ∠C_1_C_2_C_3_C_4_ vs time, and ∠C_1_C_2_C_3_C_4_ vs C_1_–C_6_ distance, respectively. In Figure 6, we see a larger dispersion of all transitions along the simulation time for FSSH than LZSH. In the latter, the transitions are distributed for 300 fs. In both cases, we notice that within the first 40 fs, most transitions are between S_1_ and S_2_ states (blue and yellow circles in Figure 6), particularly for LZSH. These facts are in agreement with the population illustrated in Figure 5a,b. Considering distances shorter than 1.75 Å (see Figure S2 in Supporting Information), we observe most transitions between S_1_ and S_2_ states for LSZH. Instead, for FSSH, there are fewer transitions between S_1_ and S_2_ states than transitions between S_1_ and S_0_ states (green and red circles in Figure 6). Interestingly, by monitoring the trajectories computed for both methods with a C_1_–C_6_ distance shorter than 1.75 Å that undergo the transitions from S_1_ to S_0_, we observe that they ended up preserving the closed ring. This agrees with the observations reported in the works by Ohta et al.^75^ and Polyak et al.^34^ on the trajectories that retained the closed ring after hitting the seam crossing between S_1_ and S_0_ with a shorter C_1_–C_6_ distance. Moreover, we notice that most trajectories with a C_1_–C_6_ distance larger than 1.75 Å undergo the transitions from S_1_ to S_0_ (see Figure S2 in Supporting Information). From Figures 7 and 8, we observe that most transitions between S_1_ and S_2_ states take place for a C_1_–C_6_ distance shorter than 1.75 Å and within the first 40 fs. For a larger C_1_–C_6_ distance, we observed that most trajectories undergo the transition from S_1_ to S_0_. In our previous study, we computed the minimum energy crossing points (MECPs) between different pairs of the electronic states involved in the reaction. For detailed information about the geometries and energies of these MECPs, refer to ref (47). At each time step, we compared these geometries with the geometry associated with the corresponding transition. We considered transitions that showed a difference in bond distance less than 0.05 Å as MECPs, which we depicted as stars. Comparing the transitions and the MECPs between S_1_ and S_2_ (full-blue stars for MECPs closer to the CHD form and pink stars for MECPs closer to the HT form), we observe that most transitions between S_1_ and S_2_ take place for geometries with shorter and longer values of the C_1_–C_6_ distance (see also Figure S2 in Supporting Information) and the torsion angle ∠C_1_C_2_C_3_C_4_. This behavior is also observed for transitions between S_1_ and S_0_ and the corresponding MECPs (green stars). Plotting the distribution of the energy difference between electronic states at hopping points along the C_1_–C_6_ distance and the torsion angle ∠C_1_C_2_C_3_C_4_ (see Figures S3 in Supporting Information), we observed clearly that the crossing seams between S_1_ and S_2_ and S_1_ and S_0_ are extended. These facts support the idea proposed by Nenov et al.^41^ about a region of the CI space that extends from the side of the closed ring to the side of the open ring.

**Figure 9:**
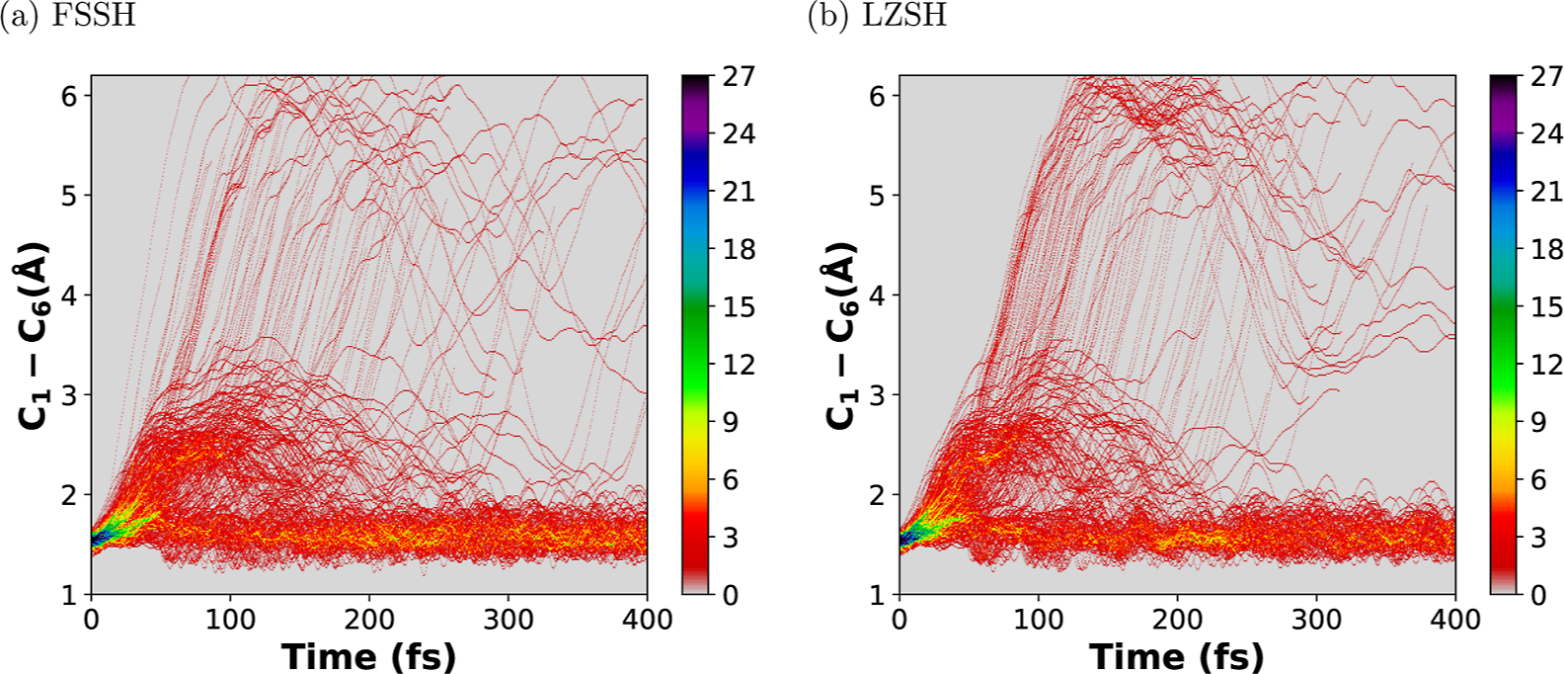
Trajectory density heat map along the simulation time of 400 fs for the C_1_–C_6_ distance for 300 trajectories. Panels (a,b) stand for the conventional Tully’s FSSH and the LZSH, respectively.

**Figure 10:**
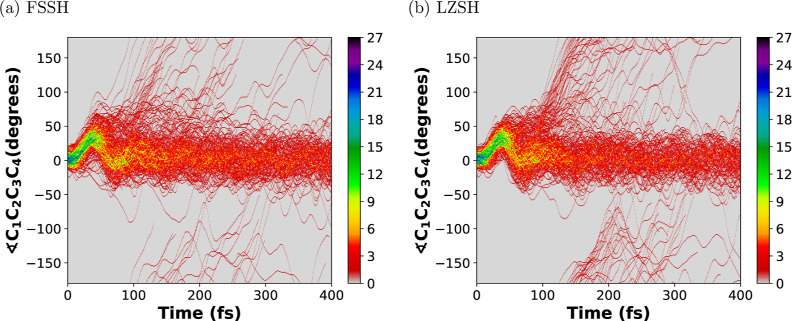
Trajectory density heat map along the simulation time of 400 fs for the torsion angle ∠C_1_C_2_C_3_C_4_ for the 300 trajectories propagated. Panels (a,b) stand for the conventional Tully’s FSSH and the LZSH, respectively.

**Figure 11:**
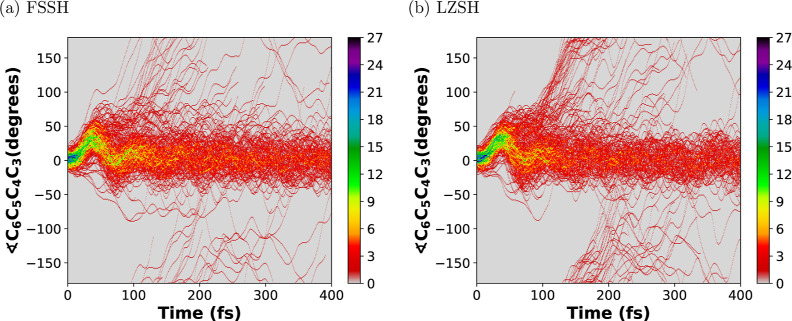
Trajectory density heat map along the simulation time of 400 fs for the torsion angle ∠C_6_C_5_C_4_C_3_ for the 300 trajectories propagated. Panels (a,b) stand for the conventional Tully’s FSSH and the LZSH, respectively.

Figures 9–11 show the trajectory density heap map time evolution for the bond-breaking coordinate (C_1_–C_6_) and the torsion angles ∠C_1_C_2_C_3_C_4_ and ∠C_6_C_5_C_4_C_3_, respectively. In Figure 9, for both methods, one can see that some trajectories (more for LZSH than for FSSH) after the first 50 fs show a large C_1_–C_6_ bond elongation. This dissociative behavior ends up in the HT photoproduct group. Other groups of trajectories oscillate around 2–3.5 Å during the first 200 fs before relaxing to either the open or closed ring. The rest of the trajectories oscillate around the equilibrium distance along the simulation time. We see that LZSH has more trajectories than FSSH that end up in the HT photoproduct. This agrees with their corresponding values of the QY formation of HT, i.e., a higher value for LZSH than that for FSSH (see Table 1). Similar oscillating behavior for the trajectories along the simulation time was reported by Polyak et al.^34^ and Schalk et al.^45^ In Figures 10 and 11, we observe that in both methods, most trajectories oscillate within a range of −25 to 50° during the first 100 fs. After that, a subset of trajectories oscillate above 50°, making some of them an entire rotation ending up in an inverted initial configuration. Notice that the torsion angles ∠C_1_C_2_C_3_C_4_ and ∠C_6_C_5_C_4_C_3_ show a similar oscillation behavior which describes a conrotatory path,^47^ i.e., the C_1_ and C_6_ rotate in the same direction. This path follows the Woodward–Hoffmann rules^76^ for a pericyclic reaction under photochemical conditions.^77^

In general, we notice that the combination of either FSSH or LZSH with SF-TDDFT can describe in good agreement the evolution of the CHD geometry with respect to those obtained by multireference wave function methods.^34,36,75^ To the best of our knowledge, nonadiabatic simulations of CHD performed using LZSH with SF-TDDFT have not been previously reported. Surprisingly, LZSH with SF-TDDFT shows results very close to those obtained by Polyak et al.^34^ and even closer by Zhang et al.^36^ using FSSH and κCSDM with the XMS-CASPT2 method, respectively. On the other hand, the combination of FSSH with SF-TDDFT shows the closest results to the experimental values.^37^ These facts show that the combination of FSSH with SF-TDDFT is more accurate than LZSH with SF-TDDFT. Thus, we observe that the lack of NACs and frustrated hops in LZSH affects its accuracy in estimating the lifetime and the QY formation of HT. At this point, it is noteworthy that LZSH calculations only require PES information for computing hopping probabilities (they do not depend on NACs) and are twice as fast as the FSSH calculations at the SF-TDDFT level of theory. Interestingly, Xie et al.^28,29^ reported that LZSH calculations are slightly 10% and twice faster than FSSH for their study on the photodissociation of pyrazine and phenol molecules, respectively. Therefore, LZSH could be an efficient method for molecular systems with weak electronic coupling. Additionally, our results suggest that SF-TDDFT can provide a good description of the shape of the adiabatic electronic states involved in the reaction. We attribute this fact to the capability of SF-TDDFT to handle double excitation,^78,79^ which is an important component in describing the topologies around CIs.^14,69^ In relation to this, our previous studies into CHD/HT photochemical interconversion using SF-TDDFT revealed that the SF-TDDFT/cc-pVDZ in combination with the BHHLYP functional was able to successfully describe critical geometries on S_0_, S_1_, and S_2_ PESs, such as different minima, ground transition state, and MECPs between S_2_/S_1_ and S_1_/S_0_. The results are in good agreement with the corresponding structures obtained by multireference wave function methods^34,80,81^ and a variant of DFT.^46^ For a detailed comparison of the PESs and CIs topologies of the aforementioned methods, refer to ref (47).

## 6 Conclusions

In the present study, we have carried out nonadiabatic dynamics simulations on the CHD molecule at the spin-flip TDDFT BHHLYP/cc-pvdz level of theory using our newly implemented surface hopping algorithm in the PySURF package based on Tully’s fewest switches approach to compare the accuracy and capability of the FSSH and the LZSH methods. Furthermore, these results were compared with the literature results at the XMS-CASPT2 level of theory. In general, LZSH at the SF-TDDFT level shows better agreement with the XMS-CASPT2 results, while the FSSH shows a closer resemblance to the experimental data. Both methods show similar tendencies for the evolution of the bond distance C_1_–C_6_ and the torsion angles ∠C_1_C_2_C_3_C_4_ and ∠C_6_C_5_C_4_C_3_ along the simulation time. Our results suggest that LZSH in combination with SF-TDDFT could be a good alternative to carrying out nonadiabatic dynamics simulations in complex systems at low computational costs in cases where the NAC vectors are not available or too expensive to compute.

## Supporting Information Available

Population dynamics, number of transitions between the electronic states, scattering plots of the energy gap, and parameters of the monoexponential fit (PDF)

## Footnotes

b A recent publication (see ref (49)) suggests that the state character of the adiabatic state S_2_ is more likely 3^1^A than 2^1^A.
