# Supplementary material for: Ultrafast Photoinduced Dynamics in 1,3-Cyclohexadiene: A Comparison of Trajectory Surface Hopping Schemes†
Source: J Chem Theory Comput. 2024 Jul 1;20(14):5796–806. doi: 10.1021/acs.jctc.4c00012 (PMC11270829; doi:10.1021/acs.jctc.4c00012)
Supplement: Supplementary file 1 — ct4c00012_si_001.pdf [file ct4c00012_si_001.pdf]

ELECTRONIC SUPPLEMENTARY INFORMATION

Ultrafast photoinduced dynamics in 1,3-cyclohexadiene: A comparison of trajectory surface hopping schemes

Edison X. Salazar,[a,b] Maximilian F. S. J. Menger[c,b] and Shirin Faraji[b,d*]

[a]Instituut-Lorentz, Universiteit Leiden, 2300 RA Leiden, The Netherlands
[b]Theoretical Chemistry, Zernike Institute for Advanced Materials, University of Groningen, Nijenborgh 4, 9747 AG Groningen, The Netherlands
[c]Theoretische Chemie, Physikalisch-Chemisches Institut, Universität Heidelberg, 69120 Heidelberg, Germany
[d] Institute of Theoretical and Computational Chemistry, Faculty of Mathematics and Natural Sciences, Heinrich Heine University Düsseldorf, 40225 Düsseldorf, Germany

ARTICLE HISTORY
Compiled June 29, 2024

Mean populations for the first 60 fs.

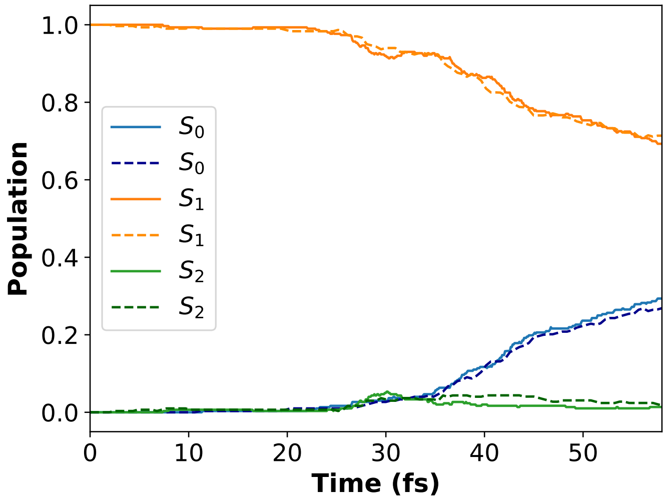

Figure S1. Population dynamics of the three lowest adiabatic electronic states (S_0 in blue, S_1 in orange, and S_2 in green) involved in the photoisomerization process of the CHD molecule for FSSH. Two sets of 300 trajectories were launched for the first 60 fs. One set used a timestep of 0.1 fs (solid lines) and the second set used a timestep of 0.5 fs (dashed lines). Notice that no significant changes are using 0.5 or 0.1 fs as timestep.

CONTACT Shirin Faraji. Email: shirin.faraji@hhu.de

Number of transitions after and before 1.75 Å.

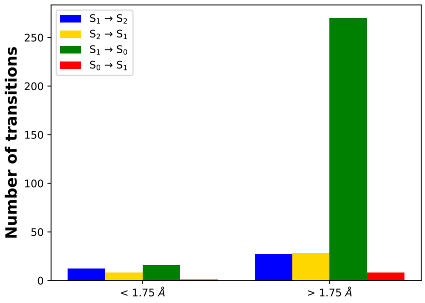

(a) FSSH

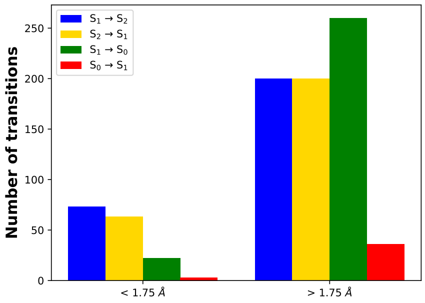

(b) FSSH′

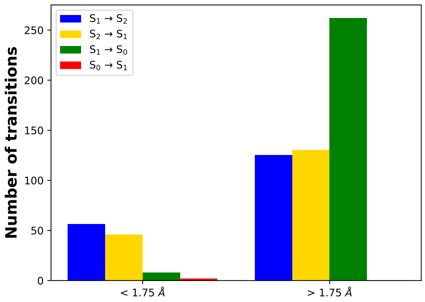

(c) LZSH

Figure S2. Number of transitions between the electronic states involved in the reaction after and before of a bond distance between the reactive carbons C_1-C_6 about of 1.75 Å. Blue, gold, green and red colors represent $S_1 \to S_2$, $S_2 \to S_1$, $S_1 \to S_0$ and $S_0 \to S_1$, respectively. Although it was observed few transitions between S_2 and S_0, we are not considering these transitions in this study because they are not providing important information for the reaction. Notice that Figure (b), FSSH′ is FSSH without considering frustrated hops constrain.

Energy gap vs C_1-C_6 bond distance.

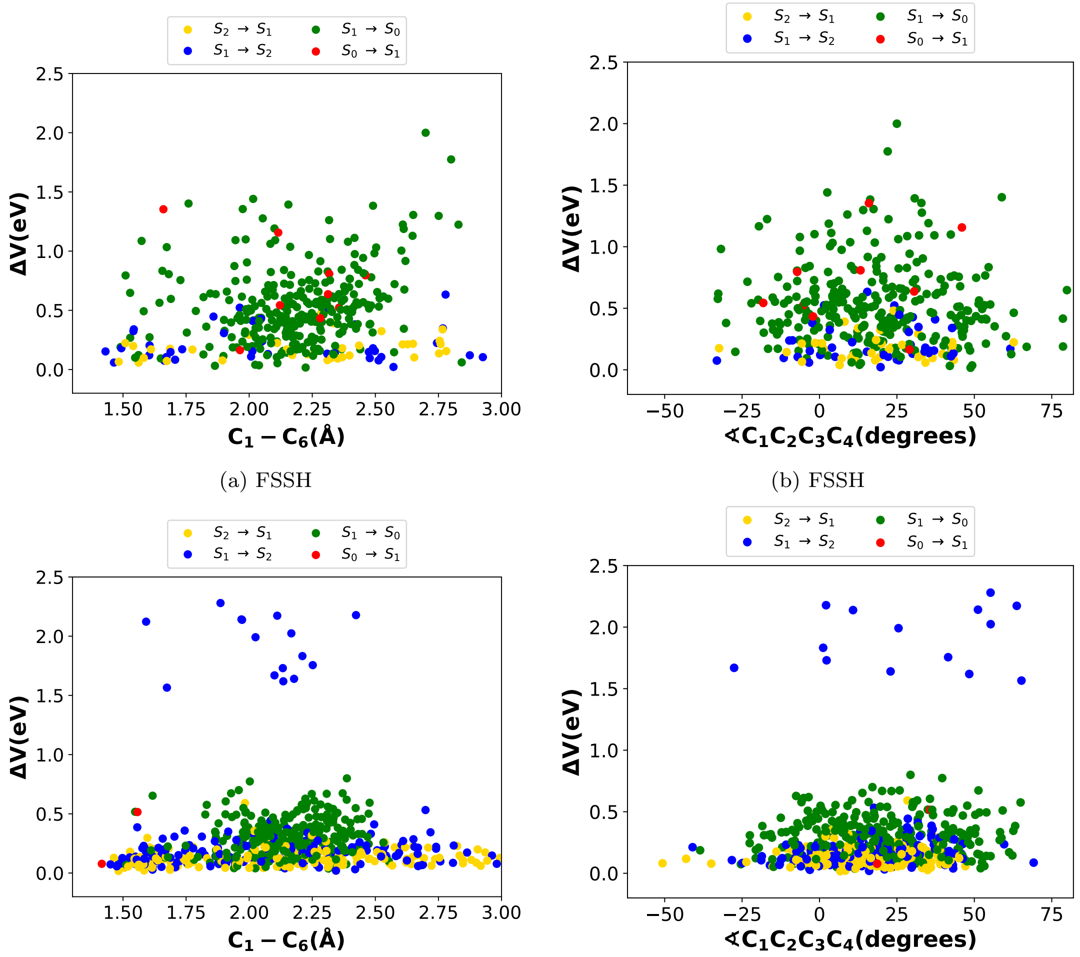

(a) FSSH

(b) FSSH

(c) LZSH

(d) LZSH

Figure S3. Scattering plots of the energy gap for $S_1 \rightarrow S_2$ and $S_1 \rightarrow S_0$ transitions as a function of the C_1-C_6 distance and the torsion angle $\sphericalangle C_1C_2C_3C_4$, respectively. Figures **(a)** and **(b)** correspond to the conventional Tully's fewest switches surface hopping (FSSH), and **(c)** and **(d)** correspond to the conventional the Landau-Zener surface hopping (LZSH).

Mean populations for FSSH, LZSH′, and LZSH.

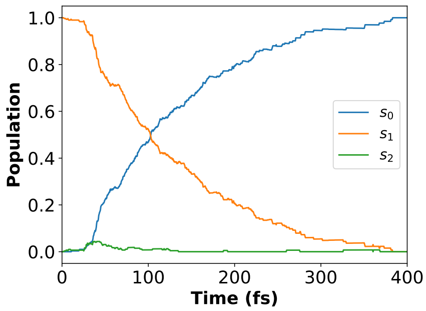

(a) FSSH

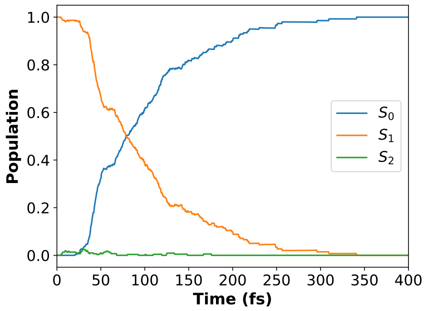

(b) LZSH′

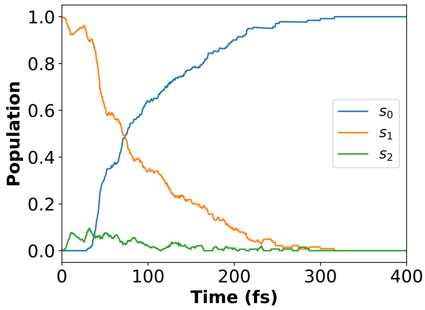

(c) LZSH

Figure S4. Population dynamics of the three lowest adiabatic electronic states (S_0 in blue, S_1 in orange, and S_2 in green) involved in the photoisomerization process of the CHD molecule for FSSH. 300 trajectories were launched with a timestep of 0.5 fs for 400 fs. Notice that Figure (b), LZSH′, is LZSH with computed NACs at the hopping points. Additionally, the velocity is corrected as in FSSH, i.e., considering frustrated hops constrain.

Table S1. Parameters of the monoexponential fit of S_1 population and QY formation of HT. τ_d represents the latency time and τ_e is the time constant for the population decay. FSSH′ is FSSH without considering frustrated hops constrain.

Methods	τ_d (fs)	τ_e (fs)	Lifetime	QY(HT)
FSSH-SF-BHHLYP/cc-pVDZ	17 ± 2	116 ± 1	133 ± 2	35 ± 5
FSSH′-SF-BHHLYP/cc-pVDZ	11 ± 2	144 ± 1	155 ± 2	26 ± 5
